# General Pathology, as Conducive to the Establishment of Rational Principles, Delivered at St. Thomas’s Hospital, during the Summer Session of 1850

**Published:** 1853-06

**Authors:** 


### REVIEWS.

---

ART. V.—*General Pathology, as conducive to the establishment of rational principles for the diagnosis and treatment of disease;* A course of lectures, delivered at St. Thomas's Hospital, during the summer session of 1850. By JOHN SIMON, F. R. S., one of the Surgical Staff of that hospital, and Officer of Health to the city of London. Philadelphia: Blanchard & Lea. 1852.

We have already noticed this work, as one of the ablest on pathology, and propose now to give a somewhat more detailed account of its contents. After some introductory considerations, as to the subject-matter, limits, and applications of pathology, its dependence on anatomical, chemical, and clinical observations; and as to disease, the import of the term, the symptoms and products, and the influence of pathology in improving its treatment, the author, in the second lecture, takes up the Blood, of which a most interesting description is given in its relations to disease. He begins by showing, that the pathology of the blood is not merely a chemical study, but is intimately related to physiology. The blood is the subject of many morbid changes, the result of causes acting from within and without the system. Morbid ingesta contaminate it; foreign bodies may be introduced into it; cancer cells, pus, and other abnormal products affect it; it becomes diseased from the retention of various excretions, as bile, carbonic acid, and urea; it is impoverished by the drain of elements that ought to be retained. Its composition is most unequivocally modified in cholera, diabetes, hemorrhages, chlorosis, and scurvy, but it would perhaps be difficult to mention a constitutional disease in which it remains unaffected; and hence the study of hematology is now attracting so much of the attention of pathologists. We have not revived the humoral pathology of our fathers, but, under the old name, are creating a science on the basis of experiment and observation. All that relates to the blood is interesting to the physician, not only as contaminated from external sources, or overladen with effete matters, or starved and attenuated by some unnatural drain; but its growth and development, and the alteration of its several elements in various diseases. Take as an example the corpuscles.

"Their *proportionate increase* constitutes the condition of plethora; and probably, for the most part, this proportionate increase co-exists with an excess in the total quantity of circulating blood; so that not only are the vessels fuller than they ought to be—not only is the arterial pulse large, the venous stream full, and the capillary network throughout the body amply injected, but further, the blood which thus fills the vascular system is in itself of a richer and more intense quality than accords with perfect health. According to the analogy of other elements of the body, you might speak of this condition as hypertrophy of the blood. So little is known hitherto of the circumstances which determine it, that I cannot venture to say anything of its causes, except negatively. I may tell you, that although starvation will counteract the tendency to its occurrence, yet it cannot be referred exclusively to the quantities of food taken daily. Of many persons living together under similar circumstances, using precisely the same diet, and taking the same exercise, a certain proportion will tend towards plethora, while the remainder will retain a lower rate of blood-development; so that we can only leave it for the present as an ultimate fact, that—as the vital power in some persons tends to build large skeletons; in others, to accumulate fat; in others, to make bulky muscles; and this, in each case, without any assignable external cause but by virtue of some law relating to the diathesis of the individual, and inherent in him from birth;—so there are others who evince a similar liability to over-growth of the blood—to its formation, namely, in quantity or quality, out of proportion to the remaining development of the body.

Visceral congestions, especially of the brain and lungs, with a disposition to spontaneous hemorrhage, are the inconveniences which have been observed to accompany this condition of the blood, and any hemorrhage which may arise under such circumstances is to be considered critical and curative in its tendency. Most of you know, as a matter of familiar observation, how often the spontaneous occurrence of epistaxis or hemorrhoids will give complete temporary relief to the symptoms of plethora; and you will remember, from my last lecture, that nothing acts so efficiently as hemorrhage in producing that dilute and attenuated condition of the blood which is the diametrical opposite of plethora. Unfortunately, we cannot always with security leave this matter in Nature's hands; for she does not invariably select the capillaries of the rectum, or of the Schneiderian membrane, for the purpose of relief; sometimes, especially after the middle of life, when the arteries are becoming atheromatous and weak, the hemorrhage will take place in the brain, or in some other important viscus, and endanger life.

The human female has a peculiar law of blood-development, which renders her a constant illustration of what I have stated to you. Duriug about thirty years of her life she forms blood enough for herself and an infant. If she be pregnant or suckling, this redundant blood-formation fulfils its purpose of nourishing her own organism and what (as regards our present argument) may be considered the parasitic organism of her infant; but if she be neither pregnant nor suckling, then you will of course readily understand that the large blood-formation, which is normal to her for those necessities, becomes excessive in their absence,—thus exactly presenting the conditions I have spoken of as those of plethora; and, like other instances of plethora, this one tends to effect its own cure by means of recurrent hemorrhage—that, namely, from the mucous membrane of the uterus, which accompanies the discharge of unfertilized ova from the Graffian vesicles, and constitutes the periodical phenomenon of *menstruation.* Interruptions of this process most commonly occur from general causes affecting the blood development, and keeping it below that abundance for which menstruation is the pre-ordained relief; and in these conditions of anæmia, whatever evils arise, are not caused by the suppression of the catamenia, but by that impoverished condition of blood which leads to their suppression; therefore such evils are the very opposite to those of plethora. But when, as occasionally happens, the blood-development is regular and ample, and the catamenia are suppressed merely because of some local disease, which renders the uterus incapable of its function, then all the general inconveniences of vascular fulness manifest themselves; and the plethora, which cannot vent itself by the legitimate channel, seeks a periodical relief at other surfaces, where it produces hæmatemesis, epistaxis, or hæmoptysis.

2. Far more frequent, however, than an increase in the proportion of the blood-corpuscles is that aberration from the standard of health which consists in their *proportionate decrease.* There is one disease which derives its visible characters from the non-development of the blood-corpuscles, and which consists essentially in that error; and, after what I have just said on the pathology of menstruation, it cannot but interest you to know that the disease to which I allude (chlorosis) is eminently one of female puberty.

In fifteen cases of idiopathic anæmia, occurring in young women, Andral found the proportion of blood-corpuscles varying, by successive degrees, from what he considers their normal standard (127) to so low a level as 30 in a thousand; and he found (precisely as would have occurred after large hemorrhages) that the water of the blood had increased just as the corpuscles had decreased, standing in one case as high as 886. In other particulars, he considered the blood healthy; but his estimate of fibrin for healthy blood is so liberal, that probably most computators would have differed from him in respect of these cases, and would have described them as presenting an excess of that material; and the organic residue of the serum is in most cases high. It is, however, quite evident, from examination of the varions analyses which have been made, that the increase of fibrin (though often observed) is not essential to constitute chlorosis; that the disease substantially consists in an atrophic condition of the blood evinced in the non-development of its characteristic cells; and that the larger proportion of albumen generally found in connection with this state may fairly be explained, where it exists, as an accidental result of the defective cell-growth—which has left the serum more saturated with organic blastematous material than it could have remained if the normal proportion of blood-cells had been developed out of it.

Few of the effects of medicine are more immediate or more remarkable than that which results in this disease from the exhibition of iron. We all know, clinically, how soon under the influence of this remedy our patients recover their natural complexion; and a chemical analysis of the blood explains this sufficiently. F. Simon gives a case, where, after a few weeks of treatment, the proportion of blood corpuscles rose from 32 to 95 in the thousand; Herberger, one where it rose from 38 to 98; Andral and Gavarret, one where it rose (in spite of two bleedings) from 46 to 95.

It is indeed very interesting to observe the effects of treatment tally so exactly with what is known of the pathology of this disease. As regards the influence of iron on the blood, we may with little hesitation describe it as a stimulant to the development of blood-cells; in which respect its action falls within a general law, (the evidence and bearings of which I shall have other opportunities of discussing with you,) that the specific stimulus of cell-growth, in every organ of the body indiscriminately, is a material identical with, or convertible into, the natural contents of the cell.

3. There is a third point relating to the morbid development of the blood-corpuscles, to which I must direct your attention, though I cannot, from my own knowledge, give you any very definite information about it. It appears that, in some diseased conditions of the spleen, the shaped elements of the blood become liable to change or to morbid admixture; sometimes large granular globules, two or three times as large as the natural-colored corpuscles, have been found in abundance in the blood; sometimes the ordinary colorless corpuscles have been found in very great excess. The recent researches of Professor Koelliker (with which, no doubt, you have been made acquainted in the Physiological Lectures) render it highly probable that the blood-corpuscles naturally undergo their dissolution in the spleen, and that an early stage of this process consists in their being collected in groups within a cell-membrane, so as to form large granular globules, similar perhaps to those which, in certain cases of spleen diseases, have been found within the stream of the general circulation; and although, since Koelliker's observations, no great progress has been made in the pathology of the spleen, yet what little has been noticed in regard of the blood, serves quite to establish the certainty that the spleen exercises some very important influence in its development; since this fluid, when observed in cases of spleen-disease, sometimes presents products apparently derived from that organ in an unfinished process of decay; sometimes, on the other hand, shows an extraordinary abundance of blood-corpuscles in an undeveloped, colorless condition. Within the last few days (through the kindness of Dr. Peacock) I have had the opportunity of examining a few drachms of blood from a child with enlarged spleen, now in the hospital, and it did not present any very obvious deviation from the ordinary characters of blood; it coagulated perfectly, and brightened by exposure to the air; microscopical examination showed an abundance of colorless corpuscles, but not such an excess of them as to appear abnormal; and, among several specimens which I examined very carefully, I found only a solitary shape which I could suppose referrible to the spleen—viz., a faintly-marked cell, containing at one side of its cavity a cluster of three or four minute reddish-brown solid rods, such as are represented in Professor Koelliker's admirable article, (*Cyclopædia of Anatomy and Physiology*, Fig. 537,) and are considered by him to be final transformations of the coloring-matter of the blood. I noticed likewise a few such clusters without being able clearly to render visible any cell-membrane around them. In respect of the not extraordinary number of colorless corpuscles observed in this case, I ought to tell you that the patient had been submitted to the course of treatment which most of all tends to diminish the relative proportion of these corpuscles, by hastening their maturation into colored blood cells—namely, to the exhibition of iron."

The irregularities in the distribution of the blood, constituting local hyperemia, or congestion, form the subject of an interesting lecture. This condition may be active or passive, and may depend upon mechanical or physiological causes. The heart cannot by any variation in its action cause the distributive inequalities of circulation,—these influences must be local. What are they? They are either:

"(1) More blood is *attracted* to the part in consequence of the molecular changes advancing there, and the hyperæmia results directly and immediately from an influence exerted by the changing tissues on the circulating blood; or (2) by indirect means, (such as the nervous system would supply,) a modification occurs in the *impulsion* or *admission* of blood, and the hyperæmia must be considered a reflex phenomenon.

Perhaps there is partial truth in both of these suppositions; but it is not possible to examine them fairly without entering somewhat on the structure and function of arteries, and considering generally the nature and extent of influence exerted by the nervous over the vascular system.

1. It is certain that the arteries (and in some animals the veins likewise) are muscular organs; that they are capable of effecting changes in their own caliber, and consequently of affecting the nutrition of parts beyond them in the circulation. The muscularity of arteries, of which John Hunter made physiological proof, is now a matter of eyesight. The greater part of what is called the middle coat of an artery consists of fibres—not, indeed, striped and encased in limitary membrane, like those of voluntary muscle, but of fibres essentially similar to those of the urinary bladder, of the intestines, of the dartos; fibres, forming a link in that chain of transition by which muscle gradually declines into contractile areolar tissue. That this tissue possesses inherent irritability has been shown again and again by its contraction under direct mechanical stimuli, and by its susceptibility to cold. The complete emptiness of arteries which we commonly find in the dead body depends on the final exertion of this contractility under the influence of the *rigor mortis*, an exertion by which their contents are propelled into the veins. Surgically, we all know how arteries contract when they are cut assunder; and, though part of this contraction is no doubt due to elasticity, and is a mere collapse from the withdrawal of that dilating force which the circulation previously exercised, yet we are able in other cases to see arteries palpably lessen their caliber, (even when they are full of blood) in consequence of mechanical irritation and exposure to the atmosphere.

Schwann performed a very neat experiment, showing the latter quality more exactly than had previously been done; though, of course, the contraction of bloodvessels under the influence of cold has been known to the Medical Profession from time immemorial. On one very hot day, furnished with some very cold water, he brought the mesentery of a living frog into the field of a microscope, and adjusted his focus to an artery of about $\frac{1}{15}$ inch diameter. He then let the cold water fall drop by drop on the membrane, and presently found the artery contracted to a third of its previous diameter. He desisted from the application, and in half an hour the artery had resumed its former size. He then again reduced it by cold; and he continued for some time thus alternately making the artery contract and letting it dilate, just as you may do with your iris in alternately turning your eye to light or shade. The experiment is a good one, because it is so precise.

The brothers Weber have demonstrated that the muscular coat of arteries is susceptible to electro-galvanic stimulation. By passing a current through small arteries (diameter $\frac{1}{80}$ to $\frac{1}{26}$ inch) in the mesentery of frogs, they have found that within a few seconds a contraction ensues, which reduces the canal of the artery to half its previous capacity; and that a continuation of the same stimulus would by degrees completely obliterate the vessel. This contraction confines itself exactly to the portion of the artery stimulated, not extending itself (if the operation be neatly performed) beyond so much of the artery as lies between the wires. The contraction does not begin suddenly at the moment of completing contact, but gradually a few seconds afterwards, and may go on augmenting itself for some time after the contact has been broken. After a little while (provided the stimulation have not been excessive), the artery recovers its former capacity, and is liable to present again the same phenomena, on a repetition of the experiment; but if the current have been too powerful or too long continued, the artery will for a while have lost its muscular tone, and will not only refuse to contract again under the current, but will yield to the pressure of the circulation, so as to present an aneurismal bulging.

Professor Paget, in the admirable lectures which he recently delivered at the College of Surgeons, drew attention to a case in which the irritability of arteries and veins may still more easily be demonstrated in warm-blooded animals. He stated that, under a very slight amount of gentle friction, any small artery and vein in the web of the bat's wing would gradually contract at the irritated point till their canals became quite obliterated, and would then gradually expand again, as in Weber's experiment, to their former or to still greater dimensions. By his kindness, I have been enabled to verify this; and I have likewise been able to observe similar changes in the mesenteric vessels of the frog.

2. With respect to nervous influence—there is unquestionable evidence that the nervous centres affect and modify the movements of the heart; and that parts receive more or less than their usual supply of blood, under influences which are purely nervous."

We recommend this treatise to all readers who wish to study Pathology in its best light. The quotations we have made will afford a very correct impression of the manner in which the learned author presents the science.

